# Novel Radiofrequency Ablation Strategies for Terminating Atrial Fibrillation in the Left Atrium: A Simulation Study

**DOI:** 10.3389/fphys.2016.00108

**Published:** 2016-04-12

**Authors:** Jason D. Bayer, Caroline H. Roney, Ali Pashaei, Pierre Jaïs, Edward J. Vigmond

**Affiliations:** ^1^Electrophysiology and Heart Modeling Institute (LIRYC), Bordeaux University FoundationPessac, France; ^2^Cardiothoracic Research Center of Bordeaux (Inserm U 1045), University of BordeauxBordeaux, France; ^3^Institute of Mathematics of Bordeaux (IMB), University of BordeauxTalence, France; ^4^Haut-Lévêque Cardiology Hospital, University Hospital Center (CHU) of BordeauxPessac, France

**Keywords:** persistent atrial fibrillation, computer modeling, phase singularity mapping, ablation, fibrosis

## Abstract

Pulmonary vein isolation (PVI) with radiofrequency ablation (RFA) is the cornerstone of atrial fibrillation (AF) therapy, but few strategies exist for when it fails. To guide RFA, phase singularity (PS) mapping locates reentrant electrical waves (rotors) that perpetuate AF. The goal of this study was to test existing and develop new RFA strategies for terminating rotors identified with PS mapping. It is unsafe to test experimental RFA strategies in patients, so they were evaluated *in silico* using a bilayer computer model of the human atria with persistent AF (pAF) electrical (ionic) and structural (fibrosis) remodeling. pAF was initiated by rapidly pacing the right (RSPV) and left (LSPV) superior pulmonary veins during sinus rhythm, and rotor dynamics quantified by PS analysis. Three RFA strategies were studied: (i) PVI, roof, and mitral lines; (ii) circles, perforated circles, lines, and crosses 0.5–1.5 cm in diameter/length administered near rotor locations/pathways identified by PS mapping; and (iii) 4–8 lines streamlining the sequence of electrical activation during sinus rhythm. As in pAF patients, 2 ± 1 rotors with cycle length 185 ± 4 ms and short PS duration 452 ± 401 ms perpetuated simulated pAF. Spatially, PS density had weak to moderate positive correlations with fibrosis density (RSPV: *r* = 0.38, *p* = 0.35, LSPV: *r* = 0.77, *p* = 0.02). RFA PVI, mitral, and roof lines failed to terminate pAF, but RFA perforated circles and lines 1.5 cm in diameter/length terminated meandering rotors from RSPV pacing when placed at locations with high PS density. Similarly, RFA circles, perforated circles, and crosses 1.5 cm in diameter/length terminated stationary rotors from LSPV pacing. The most effective strategy for terminating pAF was to streamline the sequence of activation during sinus rhythm with >4 RFA lines. These results demonstrate that co-localizing 1.5 cm RFA lesions with locations of high PS density is a promising strategy for terminating pAF rotors. For patients immune to PVI, roof, mitral, and PS guided RFA strategies, streamlining patient-specific activation sequences during sinus rhythm is a robust but challenging alternative.

## 1. Introduction

Atrial fibrillation (AF) is a worldwide epidemic that decreases the quality of life and increases mortality in its victims (Go et al., 2001). From theoretical AF studies (Lewis, 1924; Allessie et al., 1977), to animal AF models (Davidenko et al., 1990; Gray et al., 1998), to AF patients (Narayan et al., 2012; Haïssaguerre et al., 2014), AF is driven by high-frequency electrical activity that prevents the synchronized muscular contractions of the left (LA) and right (RA) atrium. The mechanism for this fibrillatory behavior can be explained by either focal impulse generation leading to the collision of multiple wavelets (Moe and Abildskov, 1959), or via spiraling reentrant waves with peripheral wave breakup (Davidenko et al., 1990). This present study focuses on the latter, where the sources of AF are termed rotors.

To extinguish AF rotors in patients, radiofrequency ablation (RFA) is used to create lesions that electrically isolate or destroy AF triggers and proarrhythmic substrates. Since its identification in the late 1990's (Haïssaguerre et al., 1998), the cornerstone of modern day AF treatment with RFA is pulmonary vein (PV) isolation (PVI) (Calkins et al., 2012), which is achieved with circular RFA lesions around the base of the right and left PVs. When AF persists post PVI, additional lesions are delivered to areas of complex fractionated atrial electrograms, or across the LA roof and/or between the left PVI and mitral value opening. PVI has an 80% success rate in patients with paroxysmal AF (Wilber et al., 2010; Morillo et al., 2014), but has little efficacy in patients with persistent AF (pAF) (Calkins et al., 2012). One explanation for the latter may be that multiple rotors can exist far from the PVs of pAF patients (Narayan et al., 2012; Haïssaguerre et al., 2014), likely being the result of extensive electrical (Nattel et al., 2008) and structural (Cochet et al., 2015) remodeling throughout the LA. Therefore, without spatial knowledge on the electrical behavior of pAF rotors in patients prior to RFA procedures, terminating pAF can be extremely challenging with long procedure times and excessive tissue damage from the many unguided RFA lesions administered to extinguish all rotors (O'Neill et al., 2009).

To improve RFA efficacy in pAF patients, the mapping of rotor phase singularities (PS) with basket catheters (Narayan et al., 2012) or non-invasive electrocardiographic imaging (ECGi) systems (Haïssaguerre et al., 2014) have been developed to help identify RFA targets that perpetuate pAF. PS mapping has revealed that either stationary rotors (Narayan et al., 2012) or multiple short-lived and/or meandering rotors (Haïssaguerre et al., 2014) underlie pAF. However, few RFA strategies have been proven to safely and effectively terminate these targets once they are identified. Thus, for this study it was hypothesized that safe and effective termination of pAF rotors can be achieved with small PS guided RFA lesions placed at locations of the LA with high PS density. When PVI, mitral, roof, and PS guided RFA lesions fail, it was also hypothesized that pAF can be terminated with evenly spaced RFA lines that streamline patient-specific LA activation sequences during sinus rhythm, which essentially forces electrical waves to propagate unidirectionally in the LA from early to late activation times.

Since it is unsafe to test these two hypotheses in pAF patients (Bohnen et al., 2011), a novel computationally efficient bilayer model of the human atria (Labarthe et al., 2014) was utilized to test the effects of PVI, roof, mitral, PS guided, and streamlined activation sequence RFA on pAF rotors in the LA. This model contains both electrical and structural pAF remodeling to simulate clinically relevant pAF and to corroborate previous AF and RFA computational modeling studies (Rotter et al., 2007; Reumann et al., 2008; Krueger et al., 2014; McDowell et al., 2015). The results from this study are intended to complement current modeling efforts toward more effective patient-specific AF therapies in the cardiac electrophysiology clinic (McDowell et al., 2012; Krueger et al., 2013).

## 2. Materials and methods

### 2.1. Atrial bilayer model

Several authors of this study recently contributed to the development of a novel bilayer model of the human atria to perform studies *in silico* (Labarthe et al., 2014). This model is computationally inexpensive (see Section 2.6) without compromising anatomical detail and electrical behavior, and was modified to accurately simulate the atrial electrophysiology of pAF patients (compare Tables 1, 2 with Lemery et al., 2007; Narayan et al., 2008; Krummen et al., 2012), which make it ideally suited to test the hypotheses for this study. The components of the modified pAF bilayer model are described below.

**Table 1:** **Activation times (ACT) in the pAF bilayer model during sinus rhythm**.

**Region**	**Avg. ACT ± SD (ms)**	**Earliest ACT (ms)**	**Latest ACT (ms)**
RA	57 ± 22	9	105
RAA	58 ± 15	23	90
SAN	9 ± 5	1	19
CS	94 ± 6	82	106
SVC	39 ± 10	18	62
IVC	NA	NA	NA
CTS	31 ± 10	16	54
PM	42 ± 12	16	77
BB	59 ± 9	41	80
LA	88 ± 20	47	135
LAA	110 ± 12	81	142
RSPV	71 ± 7	56	85
RIPV	89 ± 5	77	102
LSPV	103 ± 6	88	117
LIPV	125 ± 7	110	140

**Table 2:** **Action potential duration (APD) in the pAF bilayer model during sinus rhythm**.

**Region**	**Avg. APD ± SD (ms)**	**Shortest APD (ms)**	**Longest APD (ms)**
RA	236 ± 8	208	282
RAA	233 ± 5	212	253
SAN	300 ± 10	267	319
CS	227 ± 8	195	240
SVC	239 ± 7	216	273
IVC	NA	NA	NA
CTS	246 ± 5	228	259
PM	237 ± 6	215	257
BB	237 ± 10	192	259
LA	205 ± 11	161	237
LAA	197 ± 5	179	217
RSPV	159 ± 10	140	195
RIPV	156 ± 8	136	183
LSPV	155 ± 9	139	185
LIPV	155 ± 7	137	181

#### 2.1.1. Anatomy

As done in previous studies (Labarthe et al., 2012, 2014), the anatomy of the pAF bilayer model was constructed from segmented computed tomography (CT) scans of an adult human heart prone to pAF (Labarthe et al., 2014). The image-based atrial anatomy was then meshed with finite triangular elements, where fiber orientation was incorporated into each triangular element using a semi-automatic rule-based method that replicates the gross atrial fiber architecture of the human atria (Labarthe et al., 2012). The pAF bilayer model anatomy contains endocardial and epicardial layers connected by linear elements, along with fast conducting pathways (Bachmann's bundle, crista terminalis, pectinate muscles) and three distinct interatrial connections in order to recreate known conduction pathways in human atria. The computational mesh consists of 363,561 nodes, 718,915 triangular elements with an average edge length of 333 μm, and 143,611 linear elements with an average length of 93 μm.

#### 2.1.2. Electrophysiology

Electrophysiology parameters were finely-tuned to match monophasic action potential duration (APD) of pAF patients (Narayan et al., 2008; Krummen et al., 2012). In the cell model used to simulate human atrial myocyte membrane kinetics throughout the pAF bilayer model (Courtemanche et al., 1998), *g*_*Na*_ was doubled to produce realistic action potential upstroke velocities (Labarthe et al., 2014), *I*_*K*1_ conductance was decreased by 20% in order to provide a better match with clinically measured rate adaptation data (Krummen et al., 2012), and electrical remodeling during pAF was represented by reducing the ionic conductances of *I*_*to*_, *I*_*Kur*_, and *I*_*CaL*_ according to Courtemanche et al. (1999). Second, regional repolarization heterogeneity was incorporated following Aslanidi et al. (2011) and Seemann et al. (2006). Third, the electrophysiology of the pAF bilayer model was updated to include the PV and appendage ionic model parameters of Dorn et al. (2012). In the PV, *g*_*to*_ × 0.75, *g*_*CaL*_ × 0.75, *g*_*Kr*_ × 2.4, *g*_*Ks*_ × 1.87, *g*_*K*1_ × 0.67, and for the appendages, *I*_*Kr*_ was 1.6 fold greater in the LA vs. RA appendage. Lastly, the electrophysiology of the pAF bilayer model was modified to simulate realistic sinoatrial node (SAN) activation of the RA according to Fedorov et al. (2010) by isolating the SAN and connecting it to four different locations on the RA body. The conductivity values for each SAN connection were chosen by adjusting them until conduction propagated from the SAN to the RA, and then doubling them to ensure conduction between the SAN and RA under all pacing conditions.

All electrophysiological parameters of the pAF bilayer model used for this study are listed in Supplementary Tables S1–S3 of the online supplement, and baseline activation times (according to Section 2.4.3) and APD (at 90% repolarization) during sinus rhythm in the pAF bilayer model are listed in Tables 1, 2. Please note, activation times and APD in the inferior vena cava (IVC) of the model are not available since it is not activated during sinus rhythm. Only in rare cases is electrically active muscular tissue found within the IVCs of patients (Scave et al., 2003). In elements assigned to be non-conductive, conductivity values were set to 0.001 to prevent numerical issues from a division by zero in the conductivity tensor of the equations governing the spread of electrical activity in the pAF bilayer model.

#### 2.1.3. Fibrosis

Image-based fibrosis from patients was incorporated into the pAF bilayer model to simulate pAF consistent with other computational studies (Krueger et al., 2014; McDowell et al., 2015). Fibrotic distributions were assigned identically to the epicardial and endocardial layers, and were incorporated as microstructural discontinuities, for which mesh element edges were stochastically selected as fibrotic. This selection depended on statistical distributions of late-gadolinium enhancement, averaged across a population of pAF patients (Cochet et al., 2015), and also on the direction of a given mesh element edge compared to the longitudinal fiber direction, i.e., the direction along fibers (Labarthe et al., 2012). In order to model interstitial fibrosis, mesh element edges paralleling the longitudinal fiber direction were four times more likely to be selected than edges transversing this direction. This was implemented by using the scaling factor α(4*cos*^2^(θ) + *sin*^2^(θ)), where θ is the acute angle between a mesh element edge and the longitudinal fiber direction within the element. The constant weighting factor, α, was chosen such that the median length of connected fibrotic mesh element edges was 670 μm, which is within the range of values reported by Spach et al. (2007) for the length of interstitial fibrosis in aging fiber bundles. To determine whether to select a mesh element edge as fibrotic, each edge was assigned a uniformly distributed random number in the interval (0, 1), and then compared to the product of the scaling factor above with the late-gadolinium likelihood. Mesh element edges for which this product was greater than the random number were selected as fibrotic. In the instance that all edges for a mesh element were selected, the element was removed from the mesh. To impose no flux boundary conditions at selected edges, mesh elements edges were arranged and nodes added following the method of Costa et al. (2014).

### 2.2. pAF initiation protocol

To simulate spontaneous initiation of pAF by ectopic beats originating in the PVs (Haïssaguerre et al., 1998), pAF was initiated by rapidly pacing the right superior (RSPV) and left superior (LSPV) PVs. During sinus rhythm, which was ongoing at 86 beats per min for all simulations, each PV was paced individually for five beats with a cycle length (CL) of 160 ms and coupling interval (CI) of 240 ms at the RSPV and 400 ms at the LSPV. These values for CL and CI were taken from the clinical study by Haïssaguerre et al. (1998). pAF was considered self-sustained if it remained present for more than 10 s post initiation.

### 2.3. pAF PS analysis

An automated algorithm was used to determine the spatial distribution and duration of PSs from rotors and wavebreak. The phase of transmembrane voltage (Vm) at each computational node was calculated using the Hilbert transform of the signal, after subtraction of the mean (Umapathy et al., 2010). PSs were identified by calculating the topological charge of each element in the mesh (Rogers, 2004). PSs were tracked over time based on a distance movement threshold (Roney et al., 2015) following the network approach of Rogers (2004), with the modification that PSs rather than wavefronts were tracked over time.

PS durations were then calculated based on the birth and death of the PS, where those lasting greater than 120 ms were defined as rotors (based on the minimum pAF CL). PS and rotor density were calculated by counting the number of occurrences of a PS or rotor at each mesh element over the duration of the simulation. This score was smoothed using an inverse distance squared weighting to give an average density measure across the mesh, which was then normalized (Tzortzis et al., 2015). Metrics were also considered on a regional basis by dividing the LA into eight subdivisions, as done similarly in non-invasive PS mapping studies (Haïssaguerre et al., 2014). The subdivided LA location numbers 1–4 in **Figures 3C**, **6** correspond to posterior LA subdivisions, and numbers 5–8 correspond to anterior LA subdivisions. For regional analyses, the number of PSs and rotors in each of the eight subdivisions were calculated for every time point in the simulation (each ms), and then averaged over time in order to calculate the mean and standard deviation of PSs and rotors in each subdivision for any instance in time during the simulation.

### 2.4. Arrhythmia ablation strategies

RFA was simulated in the pAF bilayer model by setting the conductivities (longitudinal and transverse) to 0.001 S/m (non-conducting and non-zero for numerical purposes) for mesh elements pertaining to RFA lesions. Three RFA strategies were applied to the LA of the pAF bilayer model, where each is described below. Unless otherwise specified, RFA was applied 5 s post pAF initiation and analyzed over 8 s post RFA. An RFA strategy was considered to terminate pAF if it either converted pAF to atrial tachycardia (AT), i.e., a single rotor which can be terminated with specific ablation at its core (Rappel et al., 2015), or if it converted pAF back to sinus rhythm.

#### 2.4.1. RFA with PVI, roof, and mitral lines

At 5 s post pAF initiation, the right and left PVs were electrically isolated from the LA body by a continuous RFA line around each. If pAF persisted 5 s post PVI, a RFA roof line was administered between the right and left PVI lines. If pAF persisted 5 s post the RFA roof line, a RFA mitral line was administered between the left PV RFA and the mitral valve opening.

#### 2.4.2. PS guided RFA with small lesions

For each of the eight LA subdivisions used to analyze pAF rotor dynamics (**Figures 3C**, **4C**), RFA lesions were administered to each subdivision in the shape of a circle, a perforated circle to allow wavefront penetration to prevent it from becoming an anatomical obstacle, a line, or a cross. Each RFA lesion shape was tested at the following dimensions: circles from 0.5 to 1.5 cm in diameter; lines from 0.5 to 1.5 cm in length; and crosses from 0.5 to 1.5 cm in cross-sectional length. Please see the leftmost column of **Figure 6** for examples of each shape.

#### 2.4.3. Streamlining activation sequences during sinus rhythm with RFA lines

During sinus rhythm, local activation times were computed for each pAF bilayer mesh node at the instant when Vm exceeded a threshold of –40 mV. RFA lines (sets of 4–8) were then administered to the LA following the streamlines of the activation sequence. These continuous RFA lines originated at an initial point along the isochrone line 10 ms after the earliest activation time in the LA, and ended at points around the mitral value opening. The pathway of RFA lines between these points followed the gradient of the local activation time sequence, thus streamlining the activation sequence. Hence, this approach does not target individual AF targets or substrates, but aims to terminate all pAF rotors at once by limiting propagating electrical activity in the LA to follow normal activation sequences during sinus rhythm. Note, RA activation sequences were not streamlined for this study.

### 2.5. Statistical analysis

The relationships between PS density, fibrosis density, and PS guided lesion shapes and sizes in the subdivided LA were addressed by computing and analyzing Pearson product-moment correlation coefficients for every combination. Values for PS density and fibrosis density were normalized from 0 to 1 in each LA subdivision of **Figures 3C**, **4C** with respect to the maximum for all subdivisions. Values for each PS guided RFA lesion shape and size in each LA subdivision were set to 1 if it terminated pAF, and 0 otherwise. Correlation *r*-values were considered statistically significant for *p* ≤ 0.05.

### 2.6. Simulation platform

Monodomain simulations were performed using the Cardiac Arrhythmia Research Package CARP (Vigmond et al., 2008), running in parallel on two high performance dual Hexa-Core Intel Xeon X5676 nodes @ 3.06 GHz with 48 GB of memory. A time step of 20 μs was used for all simulations, which required 83 min of computation time to solve for 1 s of simulation time.

## 3. Results

### 3.1. pAF initiation and PS analysis

**Figure 1:**
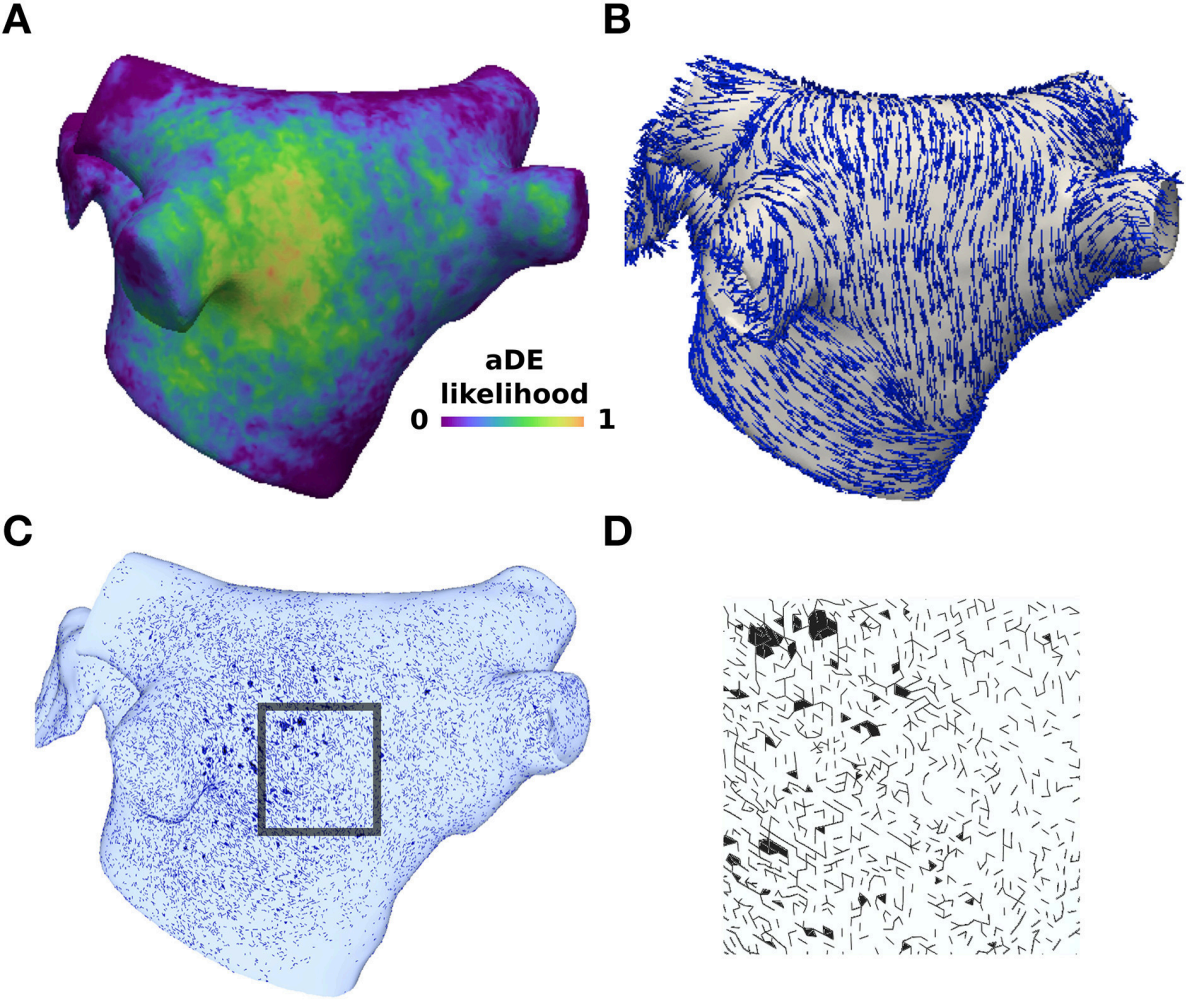
**Structural remodeling was incorporated probabilistically based on late-gadolinium enhancement data**. **(A)** Late-gadolinium enhancement likelihood data averaged across 26 patients with pAF from Cochet et al. (2015). **(B)** Longitudinal fiber directions in the pAF bilayer model. **(C)** Mesh element edges selected with probability weighted by the enhancement likelihood **(A)** and edge direction compared to the longitudinal fiber direction **(B)**. **(D)** Inset of the mesh showing element edges with no flux boundary conditions imposed to create structural discontinuities. Split mesh element edges and removed elements in **(C)** and **(B)** are marked in black.

**Figure 2:**
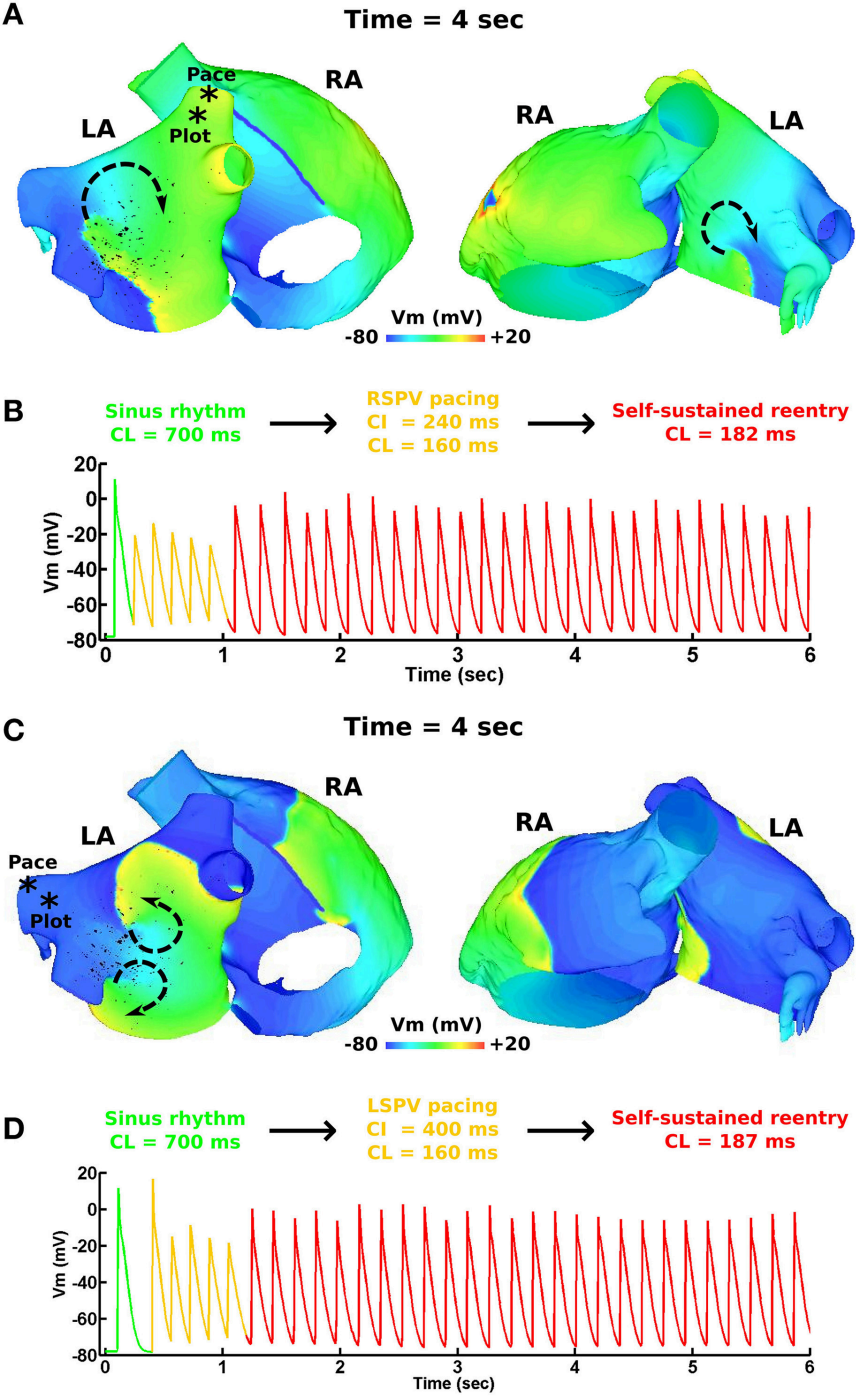
**pAF initiation via PV pacing**. **(A)** Transmembrane voltage (Vm) map post pAF initiation via RSPV pacing. **(B)** Vm plot from a point in the RSPV showing the transition of sinus rhythm to pAF via RSPV pacing. **(C)** Vm map post pAF initiation via LSPV pacing. **(D)** Vm plot from a point in the LSPV showing the transition of sinus rhythm to pAF via LSPV pacing. The * symbols in **(A,C)** indicate the pacing sites and Vm plot locations, and the arrows indicate the direction of rotor rotations.

Figure 1 demonstrates the results of applying the patient derived fibrosis to the LA of the pAF bilayer model. Self-sustained pAF was initiated in this model via the pAF initiation protocol applied to the RSPV and LSPV. pAF occurred via conduction block when the paced beats from the PVs encountered an abrupt change in fiber direction (nearly 90°) at the base of the PVs (Figure 1B) and longer APD in the LA body (160 vs. 220 ms). pAF was initiated in the same manner with fibrosis removed from this model (data not shown). Figure 2 shows examples of rotors in the fibrotic LA for both RSPV and LSPV pAF. Please see the links to Supplementary Movies 1, 2 in section 2 of the online supplement for animations of each pAF case. To summarize, multiple meandering rotors were observed on the anterior and posterior surfaces of the LA during RSPV pAF, and a figure-of-eight reentry was observed near the left inferior PV (LIPV) during LSPV pAF.

**Figure 3:**
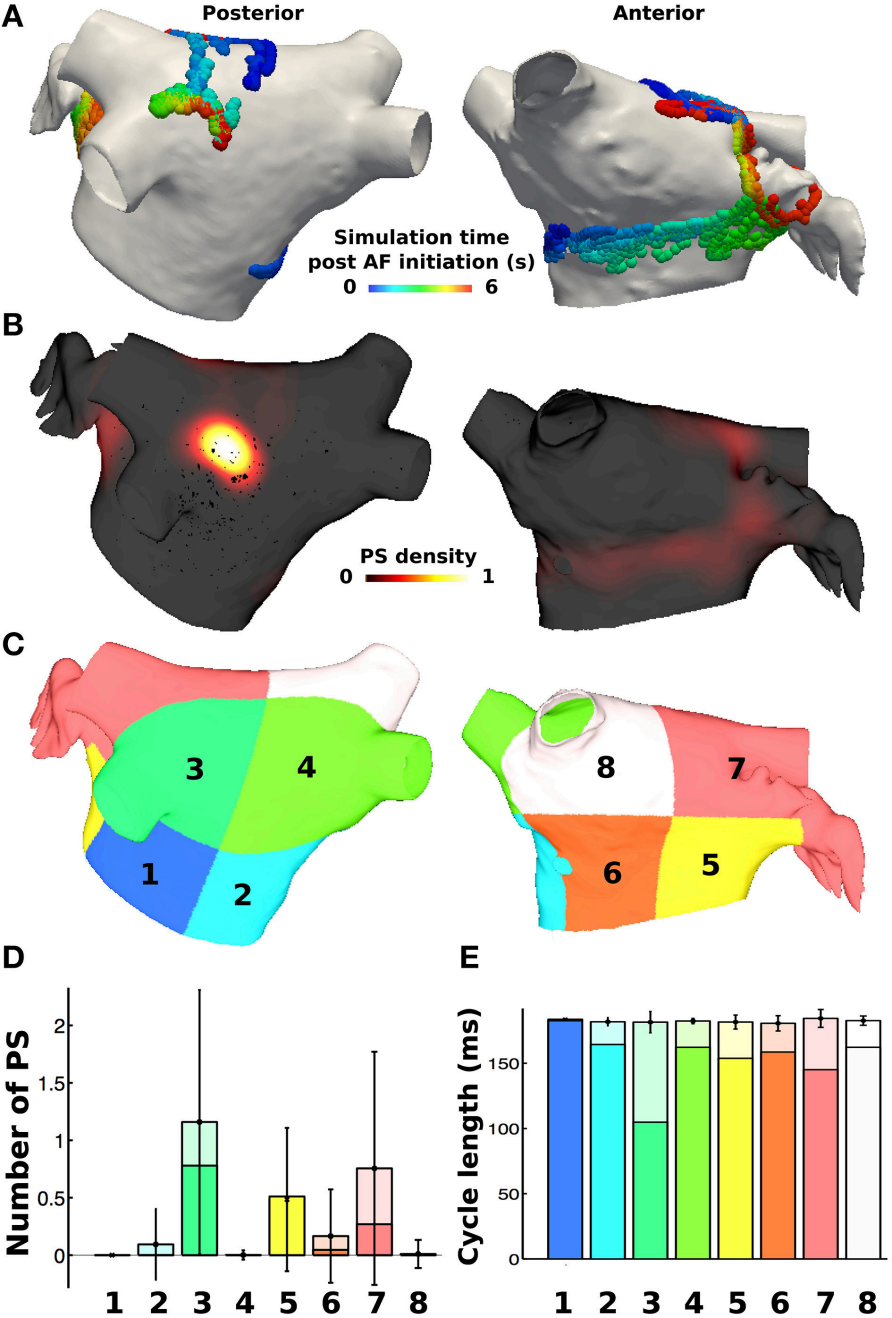
**PS locations during pAF from RSPV pacing. (A)** PS trajectories on the posterior (shown on the left) and anterior (shown on the right) walls, colored by time (ms) during the simulation post RSPV pacing. **(B)** Spatially smoothed PS density map, indicating that one rotor is found on the posterior wall in an area of high fibrosis density, and another moves across the anterior wall. **(C)** Eight LA subdivisions were used for analysis and correspond to the LA location numbers in Figure 6. **(D)** Regional assessment of the mean and standard deviation of PSs and the mean number of rotors (shown in a darker shade) over the duration of the simulation show that subdivisions 3, 5, and 7 have the highest PS density. **(E)** Regional assessment of average CL for each vertex in the pAF bilayer model indicates that mean average CL is constant across subdivisions, while the minimum average CL varies (shown in a darker shade).

**Figure 4:**
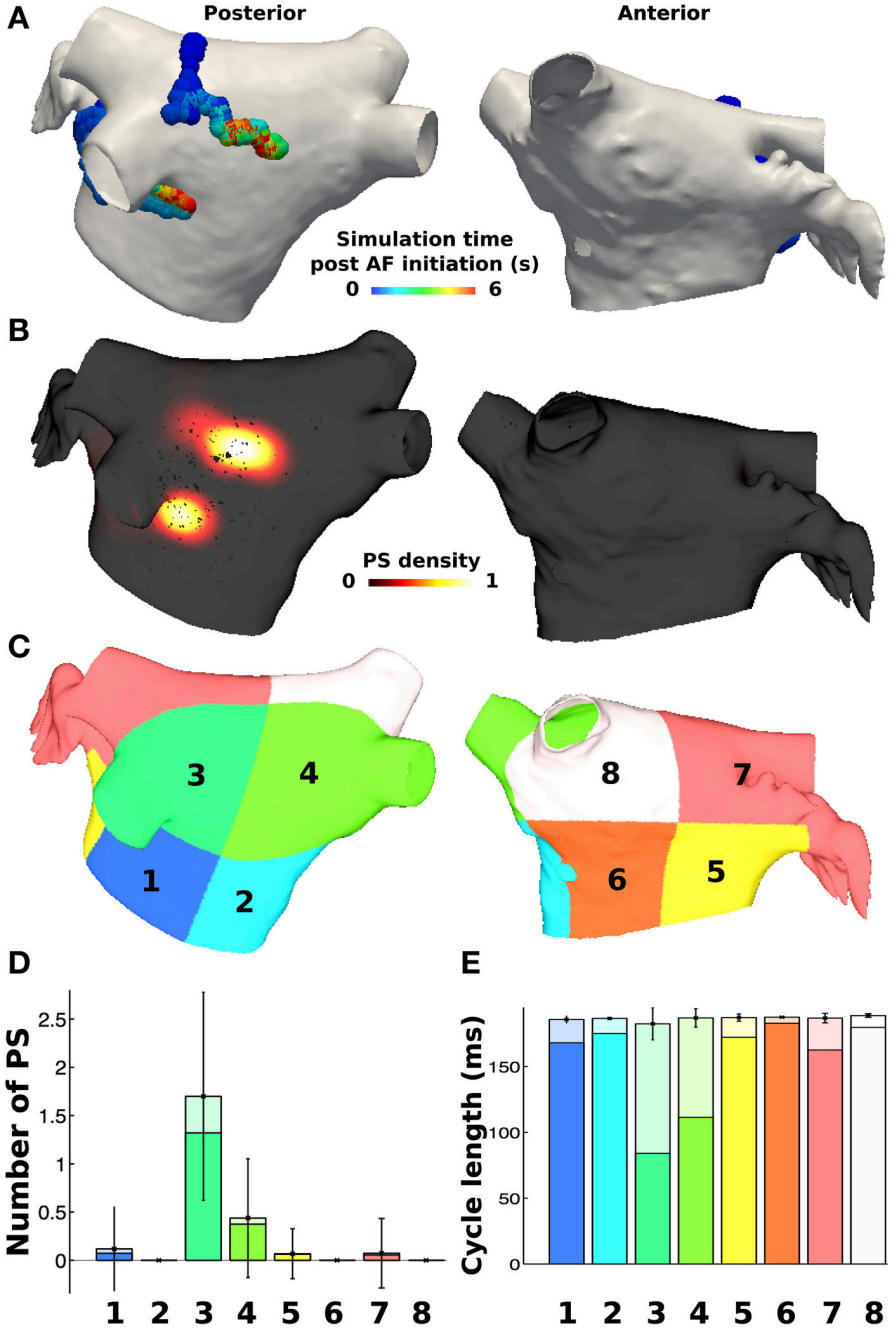
**PS locations during pAF from LSPV pacing**. **(A)** PS trajectories on the posterior (shown on the left) and anterior (shown on the right) walls, colored by time (ms) during the simulation post LSPV pacing. **(B)** Spatially smoothed PS density map, indicating rotors localized only in the area of high fibrosis density on the posterior wall. **(C)** Eight LA subdivisions were used for analysis and correspond to the LA location numbers in Figure 6. **(D)** Regional assessment of the mean and standard deviation of PSs and the mean number of rotors (shown in a darker shade) over the duration of the simulation show that subdivision 3 has the highest PS density. **(E)** Regional assessment of average CL for each vertex in the pAF bilayer model indicates that mean average CL is constant across subdivisions, while the minimum average CL varies (shown in a darker shade).

PS analysis of pAF dynamics over the entire LA was performed for the pAF in Figure 2, where the results of the analysis are provided in Figures 3A,B, 4A,B, and Table 3. For both cases of pAF, there were on average 2.0 ± 0.6 rotors with CLs of 185 ± 4 ms and PS lifespans of 452 ± 401 ms. For the LA subdivisions in Figure 3C, pAF phase analysis in Figure 3D revealed that PSs occurred in subdivisions 2, 3, 5, 6, and 7, with the highest densities in subdivisions 3, 5, and 7 corresponding to mean PSs of 1.16, 0.48, and 0.76, respectively. However, the PS density in the subdivided LA only had a weak positive correlation with fibrosis density (*r* = 0.38, *p* = 0.35). For the LA subdivisions in Figures 3C, 4C, pAF phase analysis in Figure 4D revealed that PSs occurred in subdivisions 1, 3, 4, 5, and 7, with the highest density in subdivision 3 corresponding to a mean PS of 1.7. Unlike with RSPV pAF, PS density in the subdivided LA had a much stronger positive correlation with fibrosis density (*r* = 0.77, *p* = 0.02). For reference, normalized fibrosis densities for the LA subdivisions in Figures 3C, 4C are as follows: 1 = 0.84; 2 = 0.28; 3 = 1.0; 4 = 0.57; 5 = 0.42; 6 = 0.35; 7 = 0.25; 8 = 0.20.

**Table 3:** **pAF properties in the LA**.

**Property**	**RSPV pacing**	**LSPV pacing**	**Patients**	**References**
Mean CL (ms)	182 ± 4	187 ± 4	179 ± 36	Haïssaguerre et al., 2014
Mean number of PS	2.7 ± 1.4	2.4 ± 1.0	NA	
Mean number of rotors	2.1 ± 0.9	1.9 ± 0.3	2.1 ± 1.0	Narayan et al., 2012
Mean rotor duration (ms)	419 ± 447	485 ± 355	449 ± 89	Haïssaguerre et al., 2014

For both RSPV and LSPV pAF, the highest PS densities occurred in the posterior subdivision 3 around the LIPV (Figures 3B, 4B). PS rotor trajectories on the LA surface during LSPV pAF were localized namely to LA subdivision 3, but not during RSPV pAF (compare Figures 3A, 4A with Supplementary Movies 1, 2). Furthermore, in LA subdivision 3 the LSPV pAF rotors rotated counterclockwise, while RSPV pAF rotors rotated clockwise. Therefore, spatial RSPV and LSPV pAF dynamics differed since rotors and the wavefronts radiating from them encountered electrical and structural heterogeneity in the LA differently.

### 3.2. RFA with PVI, roof, and mitral lines failed to terminate pAF

**Figure 5:**
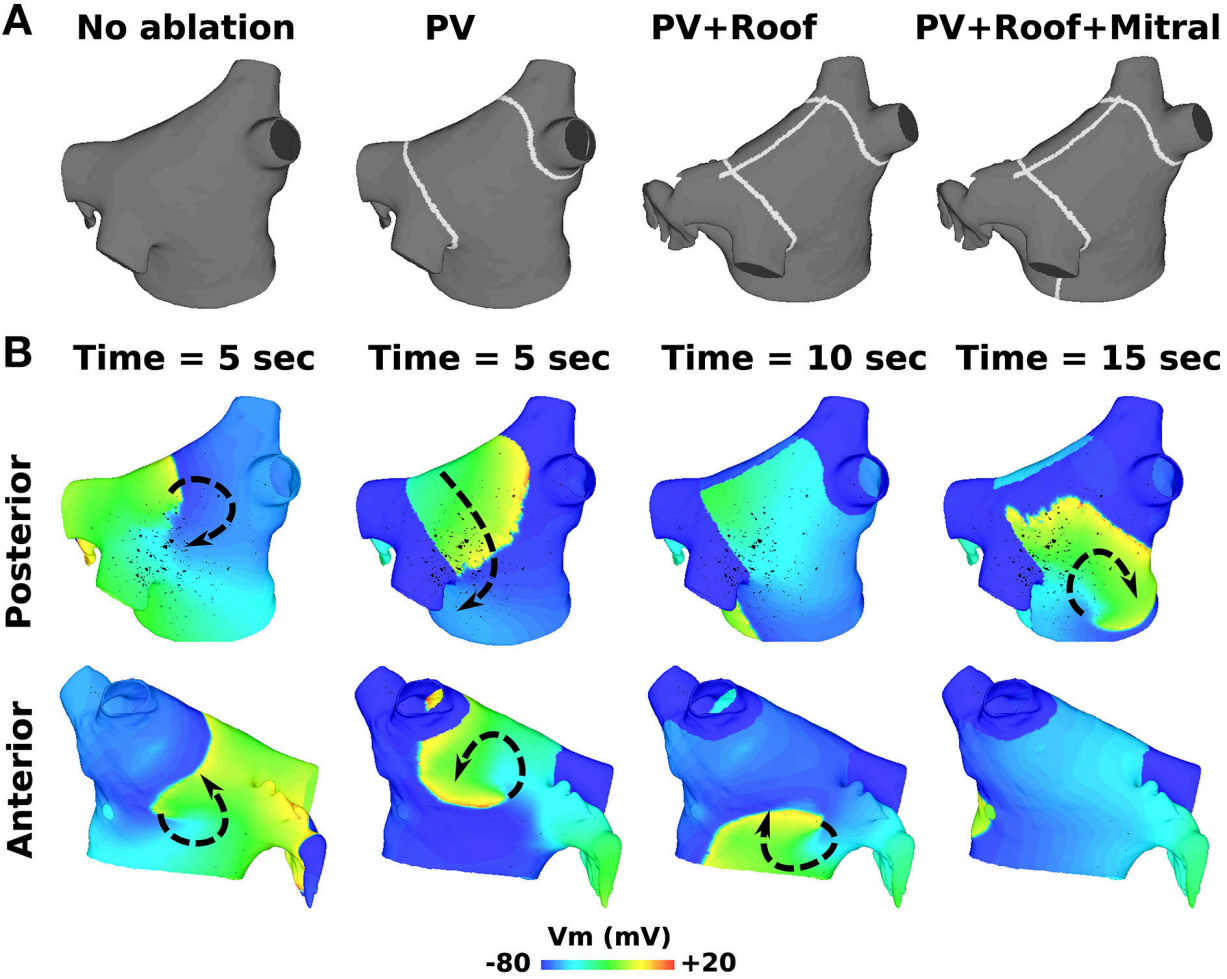
**RFA with PVI, mitral, and roof lines failed to terminate AF from RSPV pacing. (A)** RFA starting with PVI, followed by a roof line, and then a mitral line. **(B)** Transmembrane voltage (Vm) maps showing pAF rotor behavior after the respective RFA in **(A)**. The arrows indicate the direction of rotor rotations.

RFA with PVI, roof, and mitral lines failed to terminate both pAFs in Figure 2. As demonstrated in Figure 5B for RSPV pAF, PVI failed to terminate the anterior pAF rotor, which persisted even after delivering the subsequent RFA roof line. After delivering the final RFA mitral line, pAF persisted with a meandering rotor on the posterior LA body next to the LIPV. This same sequence of RFAs in Figure 5A failed to terminate LSPV pAF, and had little effect on the figure-of-eight reentry near the LIPV.

### 3.3. PS guided RFA with small lesions terminated pAF

**Figure 6:**
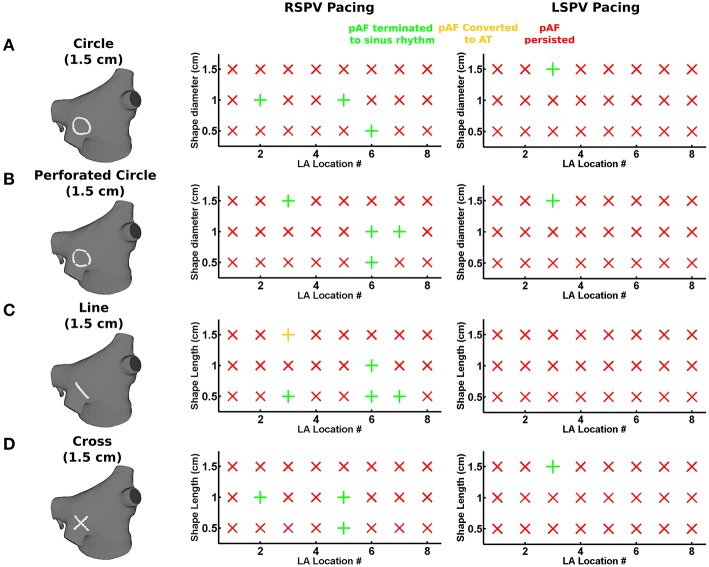
**PS guided RFA terminates pAF from RSPV and LSPV pacing**. The effects of PS guided RFA administered to the eight LA subdivision locations in Figures 3C, 4C with circles **(A)**, perforated circles **(B)**, lines **(C)**, or crosses **(D)** 1.5 cm in diameter/length on pAF initiated via RSPV or LSPV pacing: being either by terminating pAF back to sinus rhythm (green +); converting pAF to AT (yellow +); or no change with pAF persisting (red X).

**Figure 7:**
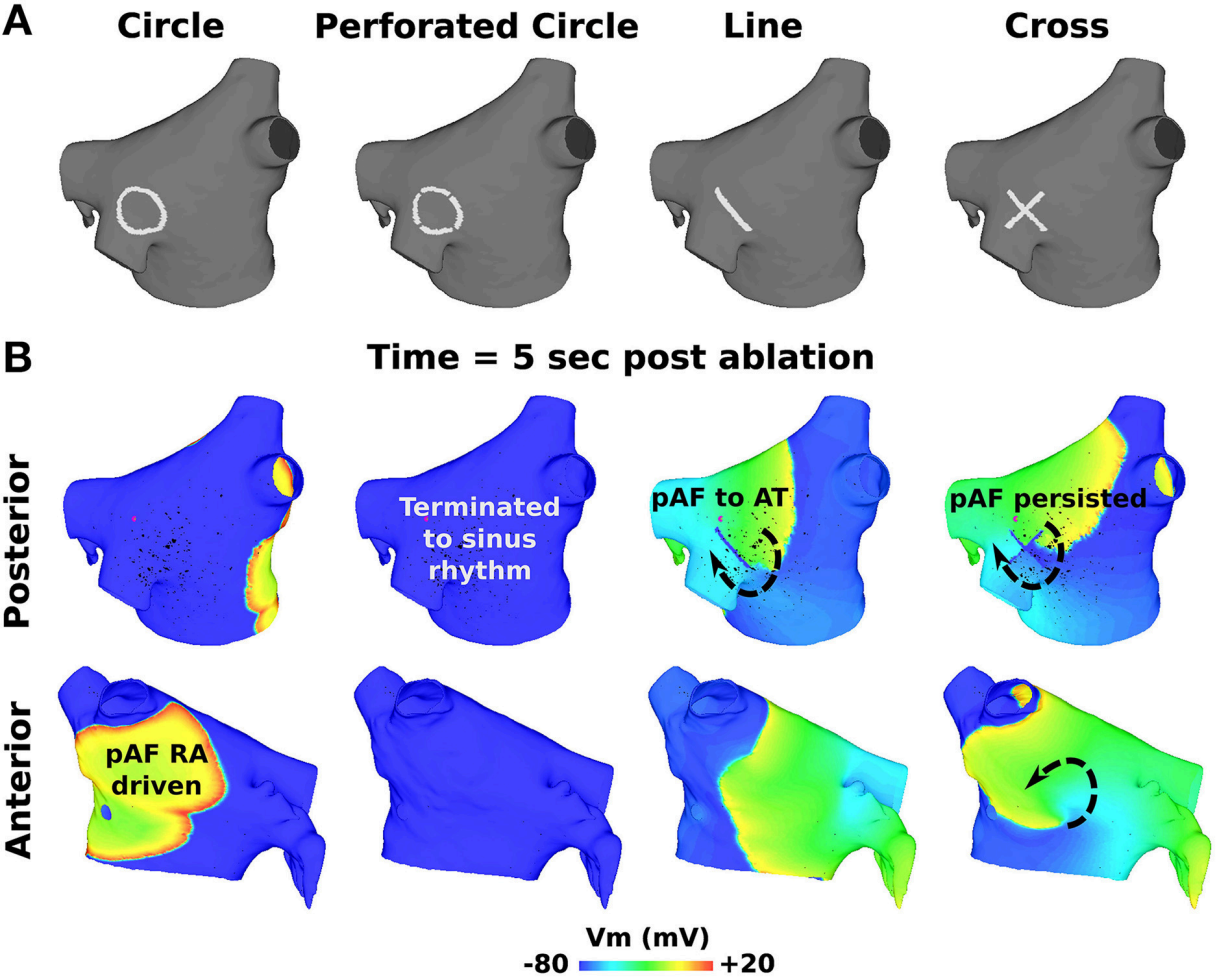
**Outcomes for PS guided RFA for pAF from RSPV pacing**. **(A)** RFA lesion shapes used for PS guided RFA with a diameter or length of 1.5 cm. **(B)** Transmembrane Voltage (Vm) maps 5 s post RFA in **(A)** showing pAF to either persist, terminate into sinus rhythm, or convert to AT. The arrows indicate the direction of rotor rotations.

Small RFA lesions delivered to locations of the LA with high PS density terminated pAF. The LA rotors either anchored to the PS guided RFA lesion to become the dominant rotor to convert pAF to AT, or it terminated the pAF completely and allowed sinus rhythm to resume. Figures 6B,C reveals that RFA with perforated circles and lines 0.5–1.5 cm in diameter/length terminated pAF when placed at LA locations with moderate to high PS density (subdivision = normalized PS density from Figure 3: 3 = 1.0; 6 = 0.14; 7 = 0.65). Figures 6A,D reveals different outcomes when placing solid circles or crosses 0.5–1.5 cm in diameter/length at LA locations with low to moderate PS density (subdivision = normalized PS density from Figure 3: 2 = 0.08; 5 = 0.42; 6 = 0.14). Figure 7 shows an example of these differences when applying each RFA lesion shape 1.5 cm in diameter/length to LA subdivision 3, where only the perforated circle and line terminated pAF, while the solid circle and cross did not. For the more stationary figure-of-eight reentry during LSPV pAF, Figures 6A,B,D reveals that RFA with circle, perforated circle, and cross lesions 1.5 cm in diameter/length terminated pAF when placed in LA subdivision 3, which had the highest density of rotors, PSs, and fibrosis. Please see the links for Supplementary Movies 3–17 in section 2 of the online supplement for animations of each successful termination of pAF from RSPV pacing, and the links for Supplementary Movies 18–20 for a few examples of failed attempts.

To quantify the results in Figures 6A–D, every combination of RFA lesion shape and size with PS and fibrosis density were tested for positive correlation. The results in Table 4 reveal PS guided with RFA perforated circles and lines had the strongest positive correlation with PS density (*r* = 0.78, *p* = 0.02) during RSPV pAF. Specifically, perforated circles 1.5 cm in diameter and lines 1.5 cm in length terminated pAF at LA locations with the highest PS density, but failed at locations of low PS density. Similar results were found for LSPV pAF, where a solid circle, perforated circle, and cross RFA lesion 1.5 cm in diameter/length had the strongest correlation with PS density (*r* = 0.97, *p* = 0.00).

**Table 4:** **Correlations of PS guided RFA lesions, fibrosis density, and PS density**.

	**Fibrosis density**		**PS density**	
	***R*-value**	***P*-value**	***R*-value**	***P*-Value**
**RSPV pacing**				
*PS density*	0.38	0.35	–	–
*RFA lesion shape and size*				
Circle 0.5 cm	−0.19	0.65	−0.16	0.70
Circle 1.0 cm	−0.29	0.48	−0.06	0.88
Circle 1.5 cm	–	–	–	–
Perforated Circle 0.5 cm	−0.19	0.65	−0.16	0.70
Perforated Circle 1.0 cm	−0.40	0.33	0.18	0.67
**Perforated Circle 1.5 cm**	0.71	0.05	**0.78**	**0.02**
Line 0.5 cm	0.13	0.77	0.69	0.06
Line 1.0 cm	−0.19	0.65	−0.16	0.70
**Line 1.5 cm**	0.71	0.05	**0.78**	**0.02**
Cross 0.5cm	−0.10	0.82	0.14	0.73
Cross 1.0cm	−0.29	0.48	−0.06	0.88
Cross 1.5 cm	–	–	–	–
**LSPV pacing**				
*PS density*	0.77	0.02	–	–
*RFA lesion shape and size*				
Circle 0.5 cm	–	–	–	–
Circle 1.0 cm	–	–	–	–
**Circle 1.5 cm**	0.71	0.05	**0.97**	**0.00**
Perforated Circle 0.5 cm	–	–	–	–
Perforated Circle 1.0 cm	–	–	–	–
**Perforated Circle 1.5 cm**	0.71	0.05	**0.97**	**0.00**
Line 0.5 cm	–	–	–	–
Line 1.0 cm	–	–	–	–
Line 1.5 cm	–	–	–	–
Cross 0.5cm	–	–	–	–
Cross 1.0cm	–	–	–	–
**Cross 1.5 cm**	0.71	0.05	**0.97**	**0.00**

*Bold values represent the maximal positive correlation between PS guided RFA results and either fibrosis or PS density*.

### 3.4. Streamlining activation sequences during sinus rhythm most effectively terminated pAF

**Figure 8:**
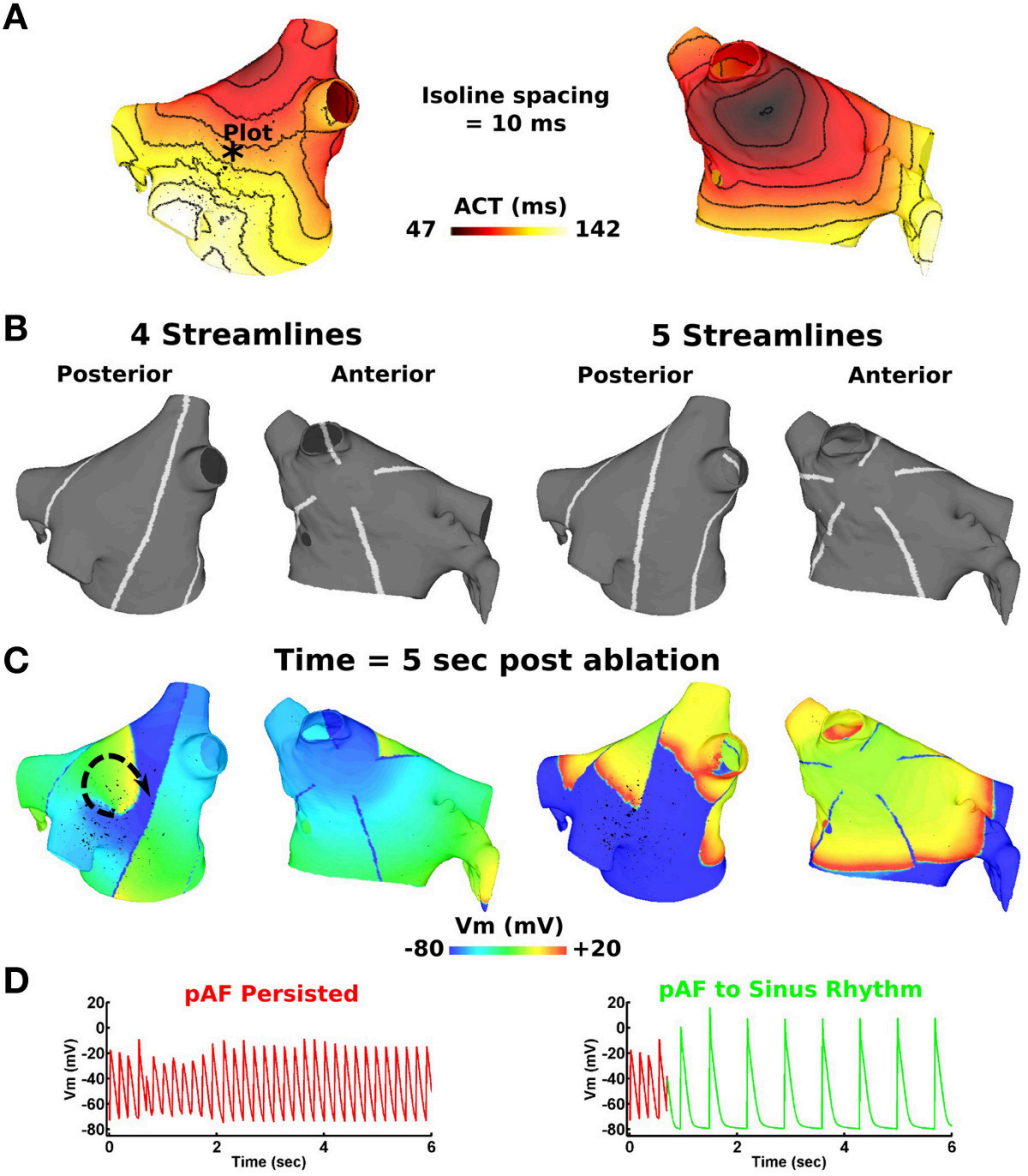
**Streamlining the sequence of LA activation during sinus rhythm with >4 lines effectively terminates pAF from RSPV pacing**. **(A)** LA activation times during sinus rhythm at 86 beats per min. **(B)** four or five RFA lines that streamline the activation time sequence in **(A)**. **(C)** Transmembrane voltage (Vm) maps 5 s post streamlining showing unsuccessful pAF termination with four RFA lines, but successful pAF termination with five RFA lines. **(D)** Vm plotted at the point indicated by the * in **(A)** before and after streamlining with four and five RFA lines.

Activation times computed during sinus rhythm in the LA (Figure 8A) reveal the earliest LA activation time to occur on the anterior LA body just below the RSPV. By administering evenly spaced continuous RFA lines that streamlined the activation time sequence in Figure 8B, RSPV pAF was immediately terminated with 5–8 RFA lines, but not with four (Figures 8C,D). Please see Supplementary Movie 21 to better visualize the rapid termination of RSPV pAF when streamlining with five RFA lines. Identical results were obtained for LSPV pAF.

## 4. Discussion

A computer model of pAF in the human LA was used to study both existing and novel RFA strategies for terminating AF. With this approach, it was determined that RFA with PVI, roof, and mitral lines failed to terminate pAF rotors far from the PVs, and likewise for small RFA lesions placed at LA locations not intersecting pAF rotor pathways. In contrast, PS guided RFA lesions 1.5 cm in diameter or length more effectively terminated pAF when placed at LA locations of high PS density. In the case that pAF rotors cannot be terminated with a combination of PVI, roof, mitral, and small PS guided RFA lesions, streamlining the sequence of electrical activation in the LA with > 4 continuous RFA lines immediately terminated pAF. The results from this study support the hypotheses that small RFA lesions guided by PS maps is a safe and effective pAF therapy, and that streamlining activation sequences during sinus rhythm with continuous RFA lines is an alternative pAF termination approach when all else fails. The clinical relevance, impact, and translation of this study's results are discussed below.

### 4.1. Corroboration of simulated and patient pAF

There is much debate as to whether pAF rotors identified with PS mapping using basket catheters have stationary rotor PSs (Narayan et al., 2012), or meandering/short-lived rotor PSs as detected non-invasively with ECGi PS mapping systems (Haïssaguerre et al., 2014). The results from this study show that both cases are feasible under different circumstances. When initiating pAF from rapidly pacing the RSPV, multiple rotors existed and meandered, rather than staying at locations of the LA with the highest fibrosis density. In contrast, when initiating pAF from rapidly pacing the LSPV, rotors lingered at the location of the pAF bilayer model with the highest fibrosis density, thus appearing stationary. In either case, pAF rotor PS statistics in the pAF bilayer model (Table 3) mirrored those obtained from clinical PS maps (Narayan et al., 2012; Haïssaguerre et al., 2014), which is compelling evidence that the pAF bilayer model was able to simulate clinically relevant pAF in order to properly test this study's hypotheses.

Secondly, terminating pAF rotors with RFA in the pAF bilayer model was difficult without a robust strategy devised *a priori*. This is consistent with the normal everyday challenges cardiac electrophysiologists encounter with RFA procedures. Particularly, the RFA with PVI, roof, and mitral lines failed to terminate pAF when pAF rotors existed far from the PVs (Figure 5), as well as failure with small RFA lesions placed at LA locations with low PS density (compare LA subdivisions 1, 4, and 8 in Figures 3C,D with Figure 6 and Table 4). Based on these results, the difficulty of PVI, roof, mitral, and small unguided RFA lesions to consistently terminate pAF rotors in all patients is unsurprising. Therefore, the novel RFA strategies developed from this study, both the small RFA lesions guided by PS mapping and the RFA lines that streamline activation sequences during sinus rhythm, are intended to provide clinicians with more strategic tools to effectively extinguish rotors that perpetuate pAF.

### 4.2. The role of fibrosis in the termination of pAF rotors

In a recent study by Chrispin et al. (2015), they found a lack of regional association between late gadolinium enhancement and pAF rotors in the LA of pAF patients. Based on these results, they concluded that fibrosis density is not guaranteed to correlate with the location of pAF rotors. In this study, a definitive positive correlation between PS and fibrosis density was not observed (see Table 4). Therefore, rather than targeting fibrosis density for RFA, it was more advantageous to target locations in the LA with the highest PS density over time, which did not always coincide with LA locations with the highest fibrosis density (compare Figure 1 and Figure 3).

A possible explanation for the inconclusive cause and effect relationship between fibrosis and PS density may be that specific fibrotic architectures are needed to generate or anchor pAF rotors. Therefore, when fibrosis density is high, the existence of unique fibrotic patterns that generate or anchor pAF may increase, but is not necessarily guaranteed. This line of thought supports the notion that patient-specific modeling of fibrosis is a critical tool to help guide AF therapies in the cardiac electrophysiology clinic (McDowell et al., 2013).

Nevertheless, one thing that is certain from these studies is that the distribution of fibrosis alters pAF rotor dynamics. When repeating the pAF initiation protocol in the pAF bilayer model with a more sparse and less dense distribution of fibrosis from a single patient in Cochet et al. (2015), PS analysis revealed significantly different pAF rotor dynamics (compare Figures 1C,D, 3, 4 with the Supplementary Figures 1, 2). These results are consistent with other computational modeling studies of AF and fibrosis (Krueger et al., 2014; McDowell et al., 2015). Though in contrast to the study by McDowell et al. (2015), PSs in this study were not always anchored and stationary. Therefore, placing RFA lesions directly over PSs did not always terminate pAF (compare Figure 3 and Figure 6), which is common behavior for RFA lesions applied to unanchored rotor PSs (Rappel et al., 2015).

### 4.3. Clinical impact and translation of the novel RFA strategies

In the clinical setting, RFA is typically performed first to terminate all rotors maintaining pAF, followed by pAF inducibility tests to identify pAF triggers for further RFA. Thus, RFA strategies for the former should be timely, effective, and minimally destructive. To help clinicians achieve these goals, two novel RFA strategies were developed *in silico* for extinguishing pAF rotors identified with clinical PS mapping systems. The clinical impact and translation of each to the bedside are discussed below.

#### 4.3.1. Small RFA lesions guided by PS mapping

This study demonstrates that terminating pAF is feasible with single RFA lesions administered to atrial locations with high PS density. Therefore, by investigating pAF rotor dynamics in PS maps from patients prior to an RFA procedure, this study predicts that targeting atrial locations with the highest PS density with small RFA lesions 1.5 cm in diameter/length will more effectively terminate rotors driving pAF in patients. In comparison to RFA strategies utilizing traditional low-resolution contact mapping (O'Neill et al., 2009), guided strategies with high-resolution PS mapping formulated before the RFA procedure could help cardiac electrophysiologists minimize RFA tissue damage and procedure times to preserve atrial function.

The technology needed to translate this PS guided RFA approach to the bedside is already in place. PS mapping systems compatible with atria imaged with CT and magnetic resonance imaging (MRI) have been cleared for clinical use and are available on the commercial market (www.cardioinsight.com). Likewise, conventional RFA catheters to deliver simple lines made of contiguous spot lesions, as well as perforated or contiguous circular lesions, are also commercially available (BW, nMARQ). Therefore, it is feasible that clinical trials could be conducted in the near future to validate the PS guided RFA results obtained from this *in silico* study.

Another technical issue that needs to be addressed is the combination of PS guided RFA lesions with PVI to isolate pAF triggers. In particular, does the order of the RFA alter pAF rotor dynamics? It is feasible that PS density distributions in the LA change significantly after each RFA administered to the patient. Therefore, real-time PS mapping may need to accompany PS guided RFA strategies in the clinic in order to obtain real-time PS maps that continually update the clinician with RFA targets.

The last technical issue to discuss is in regard to the level of precision needed for a clinician to target specific PS in the complex LA geometry. In the case that RFA locations in this study are difficult for clinicians to achieve with traditional X-ray setups, millimeter spatial precision could be achieved with interventional MRI systems for cardiac electrophysiology (Bhagirath et al., 2015). There is a growing market for MRI compatible catheters, defibrillators, etc. to make this possible in the near future.

#### 4.3.2. RFA lines streamlining the sequence of activation during sinus rhythm

This one-size-fits-all strategy developed for terminating pAF rotors with RFA lines that streamline the sequence of activation during sinus rhythm was inspired by the Cox-Maze procedure (CMP) (Cox et al., 1991). The first CMP was performed in 1987 using a cut-and-sew technique to generate a maze with lanes smaller than the critical mass for stable rotors (Garrey, 1914), thereby terminating and preventing macroreentrant pAF circuits. Similar to the CMP, this novel strategy for streamlining atrial activation sequences during sinus rhythm with RFA lines subdivides the atria into sections too small to harbor pAF rotors, which eliminates the need to target individual pAF locations and substrates.

Today, many variations of the CMP exist, which are less invasive by substituting incisions with RFA or cryoenergy lesions when possible, and are incredibly effective at terminating pAF rotors (85% pAF free at 10 year followup) (Weimar et al., 2012). However, CMPs remain prone to surgical complications due to their complexity and invasiveness when compared to RFA only procedures. CMPs also carry the risk of excessive scarring and tissue destruction, which can disrupt the normal electro-mechanical function of the atria. To minimize the dangers associated with CMPs, the RFA streamlining approach does not require destructive incisions, and requires only five RFA lines in the LA to prevent the formation and maintenance of pAF rotors. In other words, the RFA streamlining approach preserves normal electrical propagation in the LA during sinus rhythm by subdividing the LA into lanes too small to harbor pAF, but not small enough to cause macroreentry from unidirectional conduction block within a lane.

Translating the RFA streamlining approach to the bedside is much more technically challenging than PS guided RFA with smaller lesions. This is due to the fact that it is technically difficult for a clinician to quickly burn long continuous lesions over wide-areas. With technological advancements in RFA catheter designs, catheter steering systems, and MRI assisted RFA, though, this challenging but robust RFA strategy could first be validated in large animal studies, and then eventually in patients. Furthermore, it will be necessary to determine the best possible approach for obtaining patient-specific electrical activation sequences during sinus rhythm, either by trusted invasive methods (PentaRay NAV catheters) or by experimental methods tracking electrical activity on the atrial surfaces with ECGi (Ghanem et al., 2005).

### 4.4. Limitations

The first limitation of this study is that RFA lesions are delivered instantaneously, not sequentially as done in the clinic. Thus, it is feasible that RFAs administered slowly over time would alter pAF dynamics and potentially the outcome of the RFA strategies. However, this is unlikely the case for the PS guided RFA strategy since the shape dimensions used were ≤ 1.5 cm in diameter/length, so the time required for clinicians to generate these lesions should be minimal. Secondly, fibrosis is modeled as an average density across an entire patient population. Thus, patient-specific distributions of fibrosis that are significantly different than the cohort average may produce different pAF initiation and termination outcomes. Subsequent studies should identify how patient-specific differences in fibrosis density and spatial heterogeneity influence the outcome of the RFA strategies. Third, fibrosis is modeled via mesh element edge splitting rather than changes in cellular ionic properties (McDowell et al., 2015) or low conductivity (Krueger et al., 2014), which could alter pAF rotor dynamics. However, it us unknown how each of the three methods, or the combination of, most accurately models fibrosis, which warrants a future comparison study. Fourth, the way the LA was subdivided may bias the pAF and PS guided RFA analyses. For example, if a high PS density is detected in the LA appendage, RFA lesions applied to the body and not the appendage within LA subdivision 7 may have a lower chance of terminating pAF, thus weakening the positive correlation of pAF termination with PS guided RFA lesions and PS density. Future work will explore if finer subdivisions of the LA are needed. Fifth, variations in atrial wall tissue thickness (Ho et al., 1999) may affect pAF rotor dynamics (Yamazaki et al., 2012). In the current study, all layers in the pAF bilayer model have uniform thickness. If the need for specific and/or varied layer thickness arises in prospective studies, it can be easily added on an element by element basis in the pAF bilayer modeling methodology (Labarthe et al., 2014). Sixth, the atrial cell model used for this study (Courtemanche et al., 1998) is extremely sensitive to changes in the maximal conductance of *I*_*K*1_. To avoid unrealistic shortening of APD in the pAF bilayer model, which would alter pAF rotor dynamics, large increases in *I*_*K*1_ from pAF as modeled by other groups (Colman et al., 2013) were omitted from this study. Lastly, the pAF bilayer model does not include mechanical contraction which may alter pAF rotor dynamics, but the effects of mechanical contraction (quiver during pAF) on pAF rotor dynamics remains largely unknown.

## 5. Conclusion

RFA strategies for terminating pAF rotors have vastly different outcomes *in silico*. This study demonstrates that RFA with PVI, roof, and mitral lines fail to terminate pAF when rotors are far from the PVs, and likewise for small RFA lesions placed at LA locations with low PS density. In contrast, circle, line, and cross RFA lesions 1.5 cm in diameter/length terminate pAF when guided to locations of the LA with high PS density. In the case that PVI, roof, mitral, and PS guided RFA lesions fail to extinguish rotors perpetuating pAF, streamlining the sequence of LA activation during sinus rhythm with > 4 RFA lines is a robust alternative strategy to immediately terminate pAF. Further study is needed to verify the clinical relevance of these promising novel RFA strategies before translating them to the bedside.

## Author contributions

All authors have made substantial contributions to the conception (JB, PJ) and/or design (JB, CR, AP, EV) of the work; acquisition (JB), analysis (JB, CR, AP), and/or or interpretation (JB, CR, AP, PJ, EV) of data for the work; drafting the work (JB, CR) and/or revising (JB, CR, AP, PJ, EV) it critically for important intellectual content. All authors have also approved the final version to be published while agreeing to be accountable for all aspects of the work in ensuring that questions related to the accuracy or integrity of any part of the work are appropriately investigated and resolved.

## Funding

This study was funded in part by the Whitaker International Program administered by the Institute of International Education, and the Lefoulon-Delalande Foundation admini-stered by the Institute of France to JB. The research leading to these results also received funding from the European Union Seventh Framework Programme (FP7/2007-2013) under Grant Agreement HEALTH-F2-2010-261057 to PJ. AP was supported by the Agence National de Recherche grant ANR-13-MONU-0004-02. All other funding for this study was provided by the grants Equipex MUSIC ANR-11-EQPX-0030 and IHU LIRYC ANR-10-IAHU-04. High performance computing platforms for this study were provided by the computing facilities MCIA (Mésocentre de Calcul Intensif Aquitain) of the Université de Bordeaux and of the Université de Pau et des Pays de l'Adour.

### Conflict of interest statement

The authors declare that the research was conducted in the absence of any commercial or financial relationships that could be construed as a potential conflict of interest.

## Abbreviations

ACT: Activation Time
aDE: Atrial Delayed Enhancement
AF: Atrial Fibrillation
APD: Action Potential Duration (ms)
AT: Atrial Tachycardial
BB: Bachmann's Bundle
CI: Coupling Interval (ms)
CL: Cycle length (ms)
CMP: Cox-Maze Procedure
CT: Computed Tomography
CTS: Crista Terminalis
ECGi: Electrocardiographic Imaging
ENDO: Endocardium
EPI: Epicardium
GB: Gigabyte
GHz: GigaHertz
IVC: Inferior Vena Cava
LA: Left Atrium
LAA: Left Atrial Appendage
LIPV: Left Inferior Pulmonary Vein
LSPV: Left Superior Pulmonary Vein
NA: Not Available
pAF: Persistent Atrial Fibrillation
PM: Pectinate Muscle
PS: Phase Singularity
PV: Pulmonary Vein
PVI: Pulmonary Vein Isolation
RA: Right Atrium
RAA: Right Atrial Appendage
RFA: Radiofrequency Ablation
RIPV: Right Inferior Pulmonary Vein
RSPV: Right Superior Pulmonary Vein
SAN: Sinoatrial Node
SD: Standard Deviation
SVC: Superior Vena Cava
Vm: Transmembrane Voltage.
