# Supplementary material for: Novel Radiofrequency Ablation Strategies for Terminating Atrial Fibrillation in the Left Atrium: A Simulation Study
Source: Front Physiol. 2016 Apr 12;7:108. doi: 10.3389/fphys.2016.00108 (PMC4828663; doi:10.3389/fphys.2016.00108)
Supplement: Supplementary file 16 [file Presentation1.pdf]

Supplementary Material: Novel radiofrequency ablation strategies for terminating atrial fibrillation in the left atrium: a simulation study

Jason D. Bayer, Caroline H. Roney, Ali Pashaei, Pierre Jaïs and Edward J. Vigmond

*Correspondence:
Author Name: Jason D. Bayer
jason.bayer@ihu-liryc.fr

1 SUPPLEMENTARY TABLES AND FIGURES

Supplementary Table 1. Maximal conductances for ionic currents (g_x) in the Courtemanche-Ramirez-Nattel ionic model Courtemanche et al. (1998) of the atrial bilayer model *without* pAF.

Anatomical region	Maximal ionic conductances (pS/pF)					
	g_{CaL}	g_{to}	g_{Kr}	g_{Ks}	g_{K1}	$g_{Kur}(V_m)$
Default	0.1238	0.1652	0.0294	0.129	0.072	x 1.0
LA	0.1238	0.1652	0.0470	0.129	0.072	x 1.0
LAA	0.1312	0.1123	0.0470	0.129	0.072	x 1.0
RAA	0.1312	0.1123	0.0294	0.129	0.072	x 1.0
CTS	0.2067	0.2115	0.0294	0.129	0.072	x 1.0
BB	0.2067	0.2115	0.0294	0.129	0.072	x 1.0
PM	0.1238	0.1652	0.0294	0.129	0.072	x 1.0
PV	0.0928	0.1239	0.0706	0.241	0.060	x 1.0

Supplementary Table 2. Maximal conductances for ionic currents (g_x) in the Courtemanche-Ramirez-Nattel ionic model Courtemanche et al. (1999) of the atrial bilayer model *with* pAF.

Anatomical region	Maximal ionic conductances (pS/pF)					
	g_{CaL}	g_{to}	g_{Kr}	g_{Ks}	g_{K1}	$g_{Kur}(V_m)$
Default	0.0371	0.0826	0.0294	0.129	0.072	x 0.5
LA	0.0371	0.0826	0.0470	0.129	0.072	x 0.5
LAA	0.0394	0.0562	0.0470	0.129	0.072	x 0.5
RAA	0.0394	0.0562	0.0294	0.129	0.072	x 0.5
CTS	0.0620	0.1058	0.0294	0.129	0.072	x 0.5
BB	0.0620	0.1058	0.0294	0.129	0.072	x 0.5
PM	0.0371	0.0826	0.0294	0.129	0.072	x 0.5
PV	0.0278	0.0620	0.0706	0.241	0.060	x 0.5
SAN	0.1238	0.1652	0.0294	0.129	0.072	x 1.0

Supplementary Table 3. Longitudinal (g_l) and transverse (g_t) tissue conductivities for the atrial bilayer models without and with pAF.

Anatomical region	Conductivity (S/m)	
	g_l	g_t
Triangular tissue elements:		
Default	0.400	0.107
SAN	0.392	0.392
SAN line of block	0.001	0.001
IVC	0.001	0.001
CTS	0.492	0.075
PM	0.698	0.030
BB	0.702	0.181
Fibrosis	Elements removed	
Ablation	0.001	0.001
Linear bilayer connections:		
CT-RA	1.054	1.054
ENDO-EPI LA	0.326	0.326
BB-ENDO LA	3.000	3.000
BB-ENDO RA	3.000	3.000
PM-RA	1.054	1.054
BB-EPI LA	3.000	3.000
SAN-SVC	1000	1000
SAN-IVC	400	400
SAN-RAA	825	825
SAN-RA	825	825

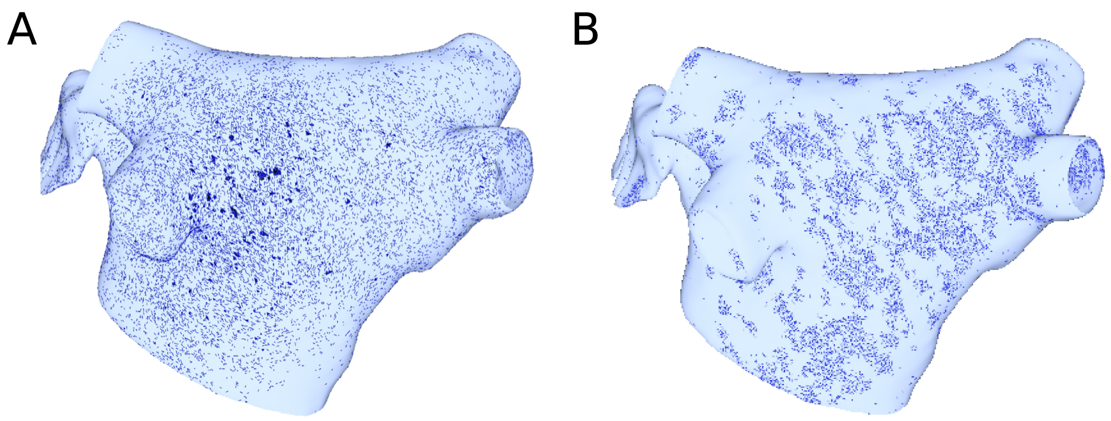

Supplementary Figure 1. *Structural remodeling was incorporated probabilistically based on late-gadolinium enhancement data.* Mesh element edges were assigned as fibrosis from late-gadolinium enhancement likelihood data being either *(A)*, averaged across 26 patients with pAF, or *(B)*, directly from a single patient with a less dense and more sparse fibrosis distribution than the averaged population Cochet et al. (2015). Split mesh element edges and removed elements in *(A)* and *(B)* are marked in black.

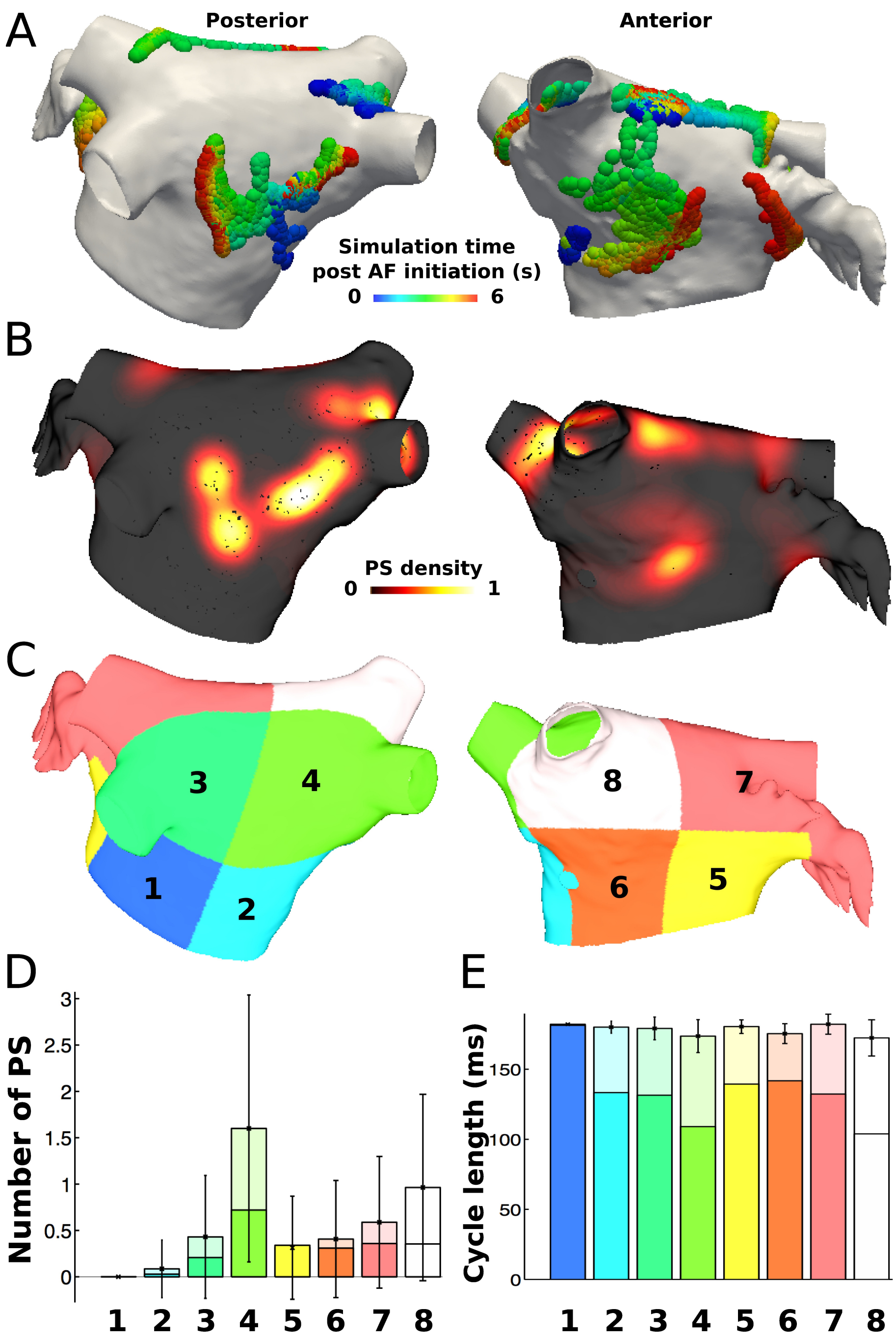

Supplementary Figure 2. *PS locations during pAF from RSPV pacing the single patient fibrosis pAF bilayer model in supplementary Figure 1.B. (A)* PS trajectories on the posterior (shown on the left) and anterior (shown on the right) walls, colored by time (ms) during the simulation post RSPV pacing. *(B)* Spatially smoothed PS density map showing the existence of multiple rotors on both the posterior and anterior walls that meandered significantly across each surface. *(C)* The eight LA subdivisions used for the analysis. *(D)* Regional assessment of the mean and standard deviation of PSs and the mean number of rotors (shown in a darker shade) over the duration of the simulation show that subdivisions 3 to 8 have comparable elevated PS densities. *(E)* Regional assessment of average CL for each vertex in the pAF bilayer model indicates that mean average CL is constant across subdivisions, while the minimum average CL varies (shown in a darker shade).

2 SUPPLEMENTARY MOVIE LIST

1. movie_1.mov: pAF initiation via RSPV pacing. This movie corresponds to Figure 2.A in the manuscript. (link)
2. movie_2.mov: pAF initiation via LSPV pacing. This movie corresponds to Figure 2.C in the manuscript. (link)
3. movie_3.mov: pAF termination with 1.0 cm circular lesion applied to LA location #2. This figure corresponds to RSPV pacing results in Figure 6.A in the manuscript. (link)
4. movie_4.mov: pAF termination with 1.0 cm circular lesion applied to LA location #5. This figure corresponds to RSPV pacing results in Figure 6.A in the manuscript. (link)
5. movie_5.mov: pAF termination with 0.5 cm circular lesion applied to LA location #6. This figure corresponds to RSPV pacing results in Figure 6.A in the manuscript. (link)
6. movie_6.mov: pAF termination with 1.5 cm perforated circular lesion applied to LA location #3. This figure corresponds to RSPV pacing results in Figure 6.B in the manuscript. (link)
7. movie_7.mov: pAF termination with 0.5 cm perforated circular lesion applied to LA location #6. This figure corresponds to RSPV pacing results in Figure 6.B in the manuscript. (link)
8. movie_8.mov: pAF termination with 1.0 cm perforated circular lesion applied to LA location #6. This figure corresponds to RSPV pacing results in Figure 6.B in the manuscript. (link)
9. movie_9.mov: pAF termination with 1.0 cm perforated circular lesion applied to LA location #7. This figure corresponds to RSPV pacing results in Figure 6.B in the manuscript. (link)
10. movie_10.mov: pAF termination with 0.5 cm linear lesion applied to LA location #3. This figure corresponds to RSPV pacing results in Figure 6.C in the manuscript. (link)
11. movie_11.mov: pAF termination with 1.5 cm linear lesion applied to LA location #3. This figure corresponds to RSPV pacing results in Figure 6.C in the manuscript. (link)
12. movie_12.mov: pAF termination with 0.5 cm linear lesion applied to LA location #6. This figure corresponds to RSPV pacing results in Figure 6.C in the manuscript. (link)
13. movie_13.mov: pAF termination with 1.0 cm linear lesion applied to LA location #6. This figure corresponds to RSPV pacing results in Figure 6.C in the manuscript. (link)
14. movie_14.mov: pAF termination with 0.5 cm linear lesion applied to LA location #7. This figure corresponds to RSPV pacing results in Figure 6.C in the manuscript. (link)
15. movie_15.mov: pAF termination with 1.0 cm cross lesion applied to LA location #2. This figure corresponds to RSPV pacing results in Figure 6.D in the manuscript. (link)
16. movie_16.mov: pAF termination with 0.5 cm cross lesion applied to LA location #5. This figure corresponds to RSPV pacing results in Figure 6.D in the manuscript. (link)
17. movie_17.mov: pAF termination with 1.0 cm cross lesion applied to LA location #5. This figure corresponds to RSPV pacing results in Figure 6.D in the manuscript. (link)
18. movie_18.mov: Failed pAF termination with 1.0 cm circular lesion applied to LA location #3. This figure corresponds to RSPV pacing results in Figure 6.A in the manuscript. (link)
19. movie_19.mov: Failed pAF termination with 1.0 cm circular lesion applied to LA location #7. This figure corresponds to RSPV pacing results in Figure 6.A in the manuscript. (link)
20. movie_20.mov: Failed pAF termination with 1.5 cm cross lesion applied to LA location #3. This figure corresponds to RSPV pacing results in Figure 6.D in the manuscript. (link)

21. movie_21.mov: pAF termination with 5 RFA activation map streamlines. This figure corresponds to RSPV pacing results in Figure 8 in the manuscript. (link)

REFERENCES

Cochet, H., Mouries, A., Nivet, H., Sacher, F., Derval, N., Denis, A., et al. (2015). Age, atrial fibrillation, and structural heart disease are the main determinants of left atrial fibrosis detected by delayed-enhanced magnetic resonance imaging in a general cardiology population. *J Cardiovasc Electrophysiol* 26, 484–492. doi:10.1111/jce.12651

Courtemanche, M., Ramirez, R. J., and Nattel, S. (1998). Ionic mechanisms underlying human atrial action potential properties: insights from a mathematical model. *Am J Physiol* 275, H301–H321

Courtemanche, M., Ramirez, R. J., and Nattel, S. (1999). Ionic targets for drug therapy and atrial fibrillation-induced electrical remodeling: insights from a mathematical model. *Cardiovasc Res* 42, 477–489
